# Supplementary material for: Autophagy-independent role of ATG9A vesicles as carriers for galectin-9 secretion
Source: Nat Commun. 2025 May 7;16:4259. doi: 10.1038/s41467-025-59605-5 (PMC12059159; doi:10.1038/s41467-025-59605-5)
Supplement: Supplementary file 1 — Supplementary information [file 41467_2025_59605_MOESM1_ESM.pdf]

Supplementary Information

Autophagy-independent role of ATG9A vesicles as carriers for galectin-9 secretion

Wenting Zhang[1+], Cuicui Ji[1+], Xianghua Li[1+], Tianlong He[1+], Wei Jiang[1], Yukun Liu[1], Meiling Wu[1], Yunpeng Zhao[1], Xuechai Chen[1], Xiaoli Wang[1], Jian Li[2], Haolin Zhang[1*], and Juan Wang[1*]

[1]College of Chemistry and Life Science, Beijing University of Technology, Beijing, 100124, China

[2]Department of Biochemistry and Molecular Biology, School of Basic Medical Sciences, Shandong University, Jinan, Shandong, 250012, China

*Correspondence: haolin.zhang@bjut.edu.cn, juanwang@bjut.edu.cn

+: Equally contributing first authors

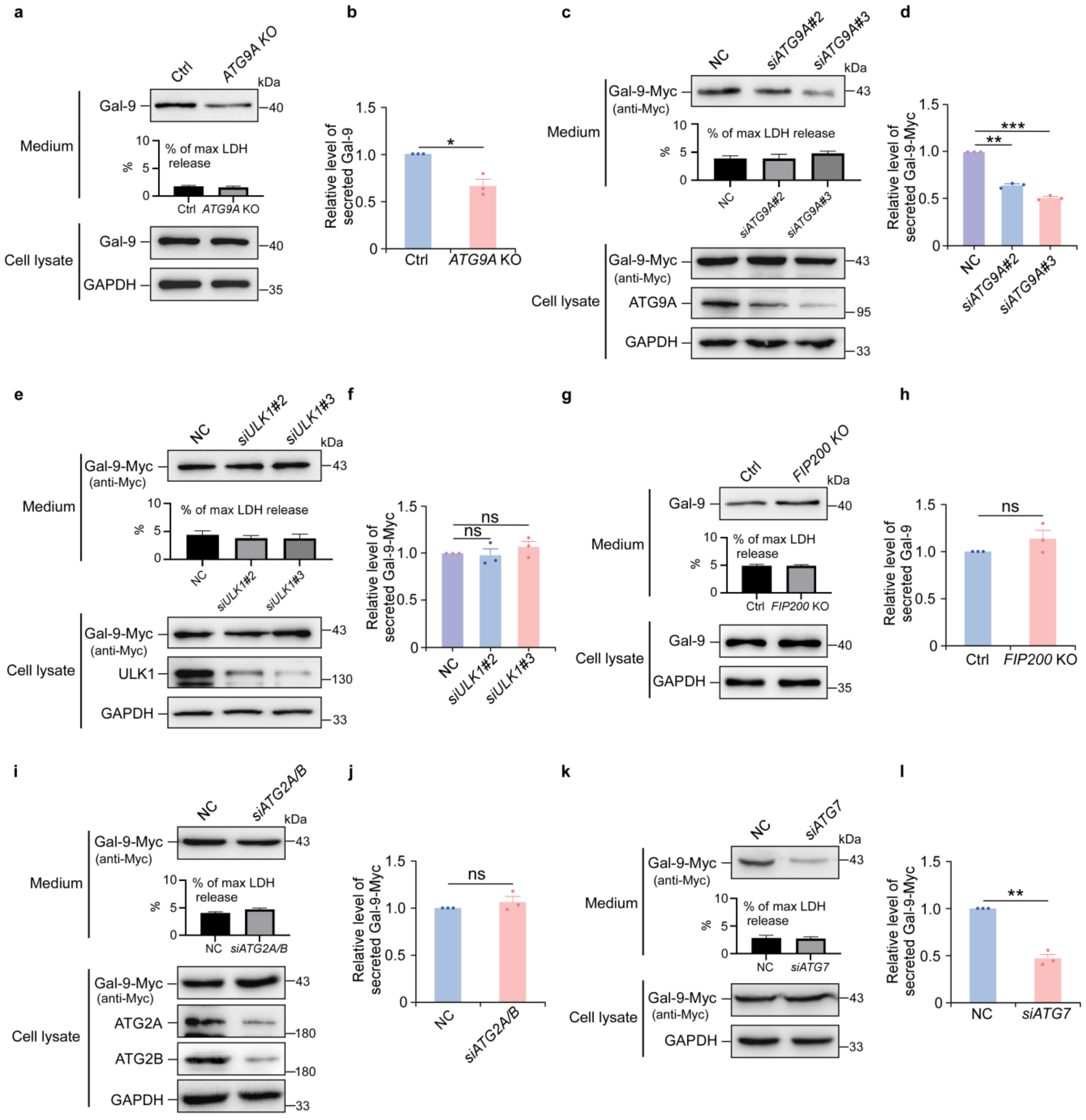

Supplementary Figure 1. Galectin-9 secretion mediated by ATG9A is independent of classical autophagy

(a, b) Galectin-9 secretion in control or *ATG9A* KO HeLa cells after 24-hour DMEM starvation was analyzed by immunoblotting (a) and quantified (b). Data are presented as mean ± SEM and analyzed using Student's t test (n = 3 independent experiments). * $p < 0.05$.

(c, d) Galectin-9 secretion in control (NC) or *ATG9A*-depleted (si*ATG9A*#2 and si*ATG9A*#3) HEK-293T cells after 24-hour DMEM starvation was analyzed by immunoblotting (c) and quantified (d). Data are presented as mean ± SEM and analyzed using Student's t test (n = 3 independent experiments). ** $p < 0.01$; *** $p < 0.001$.

(e, f) Galectin-9 secretion in control (NC) or *ULK1*-depleted (si*ULK1*#2 and si*ULK1*#3) HEK-293T cells after 24-hour DMEM starvation was analyzed by immunoblotting (e) and quantified (f). Data are presented as mean ± SEM and analyzed using Student's t test (n = 3 independent experiments). ns, not significant.

(g, h) Galectin-9 secretion in control or *FIP200* KO HeLa cells after 24-hour DMEM starvation was analyzed by immunoblotting (g) and quantified (h). Data are presented as mean ± SEM and analyzed using Student's t test (n = 3 independent experiments). ns, not significant.

(i, j) Galectin-9 secretion in control (NC) or *ATG2A/B*-depleted (si*ATG2A/B*) HeLa cells after 24-hour DMEM starvation was analyzed by immunoblotting (i) and quantified (j). Data are presented as mean ± SEM and analyzed using Student's t test (n = 3 independent experiments). ns, not significant.

(k, l) Galectin-9 secretion in control (NC) or *ATG7*-depleted (si*ATG7*) HeLa cells after 24-hour DMEM starvation was analyzed by immunoblotting (k) and quantified (l). Data are presented as mean ± SEM and analyzed using Student's t test (n = 3 independent experiments). ** $p < 0.01$.

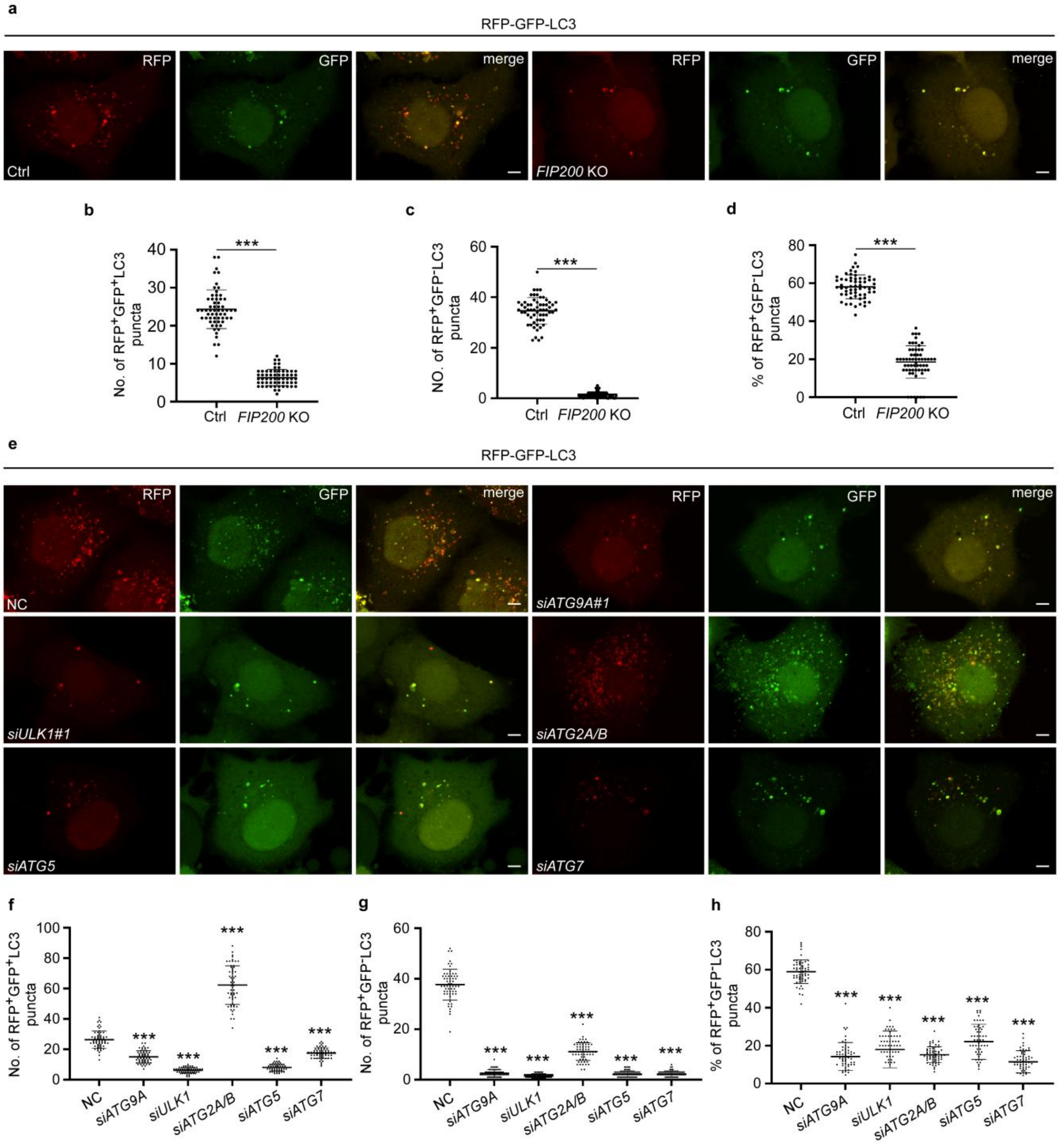

Supplementary Figure 2. Assessment of autophagic flux in cells with autophagy-related gene deletions or depletions

(a-d) Autophagic flux in control or *FIP200* KO HeLa cells analyzed using RFP-GFP-LC3 assay after 3-hour DMEM starvation. (a) Representative images. Scale bars, 5 μm. Quantification of (b) RFP$^+$GFP$^+$LC3 puncta, (c) RFP$^+$GFP$^-$ LC3 puncta, and (d) percentage of RFP$^+$GFP$^-$LC3 puncta. Data are presented as mean ± SD and analyzed

using Wilcoxon rank sum test (n = 60 cells in each group examined over 3 independent experiments). *** $p < 0.001$.

(e-h) Autophagic flux in control, *ATG9A*-depleted, *ULK1*-depleted, *ATG2A/B*-depleted, *ATG5*-depleted, or *ATG7*-depleted HeLa cells analyzed using RFP-GFP-LC3 assay after 3-hour DMEM starvation. (e) Representative images. Scale bars, 5 μm. Quantification of (f) $RFP^{+}GFP^{+}$LC3 puncta, (g) $RFP^{+}GFP^{-}$ LC3 puncta, and (h) percentage of $RFP^{+}GFP^{-}$LC3 puncta. Data are presented as mean ± SD and analyzed using Student's t test, Welch's t test and Wilcoxon rank sum test (n = 60 cells in each group examined over 3 independent experiments). *** $p < 0.001$.

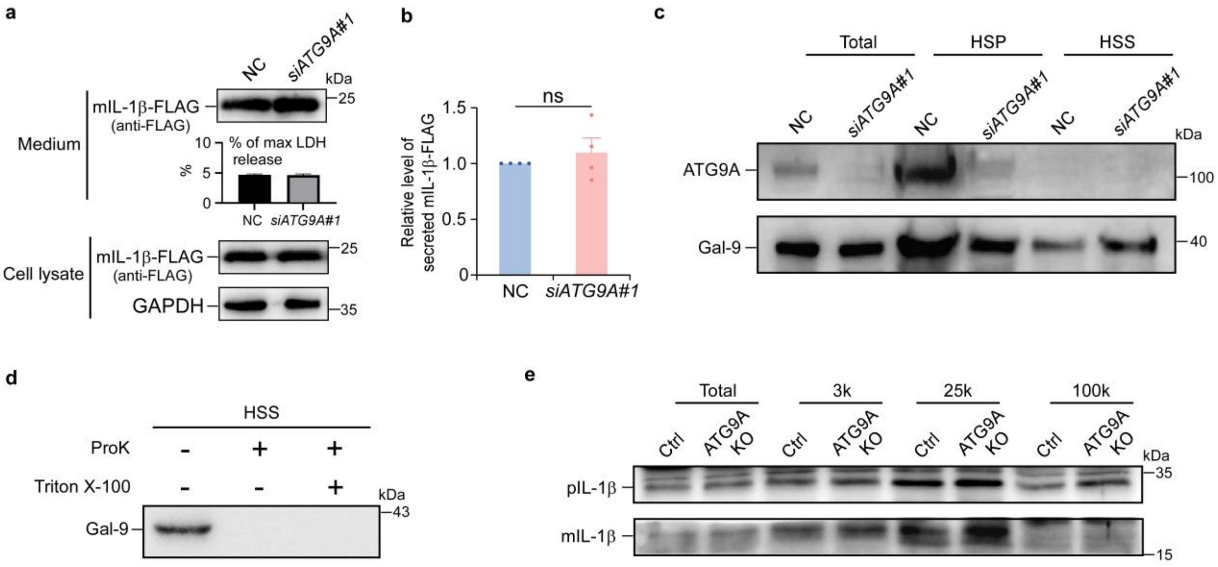

Supplementary Figure 3. ATG9A influences the membrane association of galectin-9 but has no effect on IL-1β

(a, b) Secretion of FLAG-tagged mature IL-1β (mIL-1β-FLAG) in control (NC) or *ATG9A*-depleted (si*ATG9A*#1) HEK-293T cells after 24-hour DMEM starvation was analyzed by immunoblotting (a) and quantified (b). Data are presented as mean ± SEM and analyzed using Student's t test (n = 4 independent experiments). ns, not significant.

(c) Immunoblot analysis of ATG9A and galectin-9 distribution in 100K membrane pellet fractions (HSP) and supernatant fractions (HSS) from control (NC) or *ATG9A*-depleted (si*ATG9A*#1) HEK-293T cell lysates after differential centrifugation. Representative data from three independent experiments are shown.

(d) Cell lysates from HeLa cells after 24-hour DMEM starvation were centrifuged at 100,000 × g. The HSS was divided into three fractions and treated with or without proteinase K (Pro K) and Triton X-100, followed by immunoblot analysis using an anti-galectin-9 antibody. Representative data from three independent experiments are shown.

(e) Immunoblot analysis of mature (m) and precursor (p) IL-1β in membrane fractions isolated from control (NC) or *ATG9A* KO HeLa cell lysates after differential centrifugation. Representative data from three independent experiments are shown.

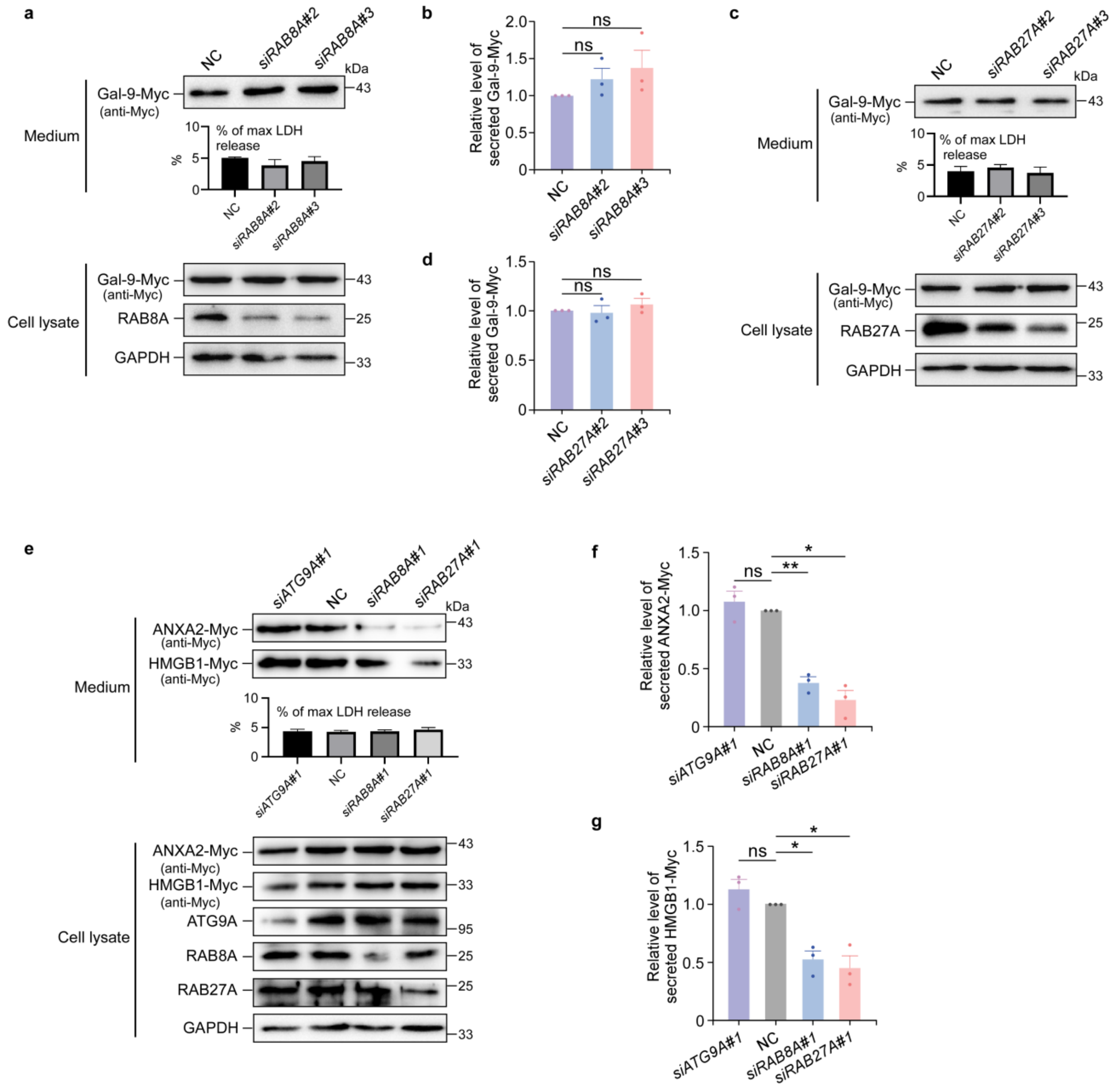

Supplementary Figure 4. Secretion of galectin-9 is independent of RAB8A and RAB27A

(a, b) Galectin-9 secretion in control (NC) or *RAB8A*-depleted (si*RAB8A*#2 and si*RAB8A*#3) HEK-293T cells starved in DMEM for 24 hours was analyzed by immunoblotting (a) and quantified (b). Secretion of indicated proteins in control cells was normalized to 1. Data are presented as mean ± SEM and analyzed using Student's t test (n = 3 independent experiments). ns, not significant.

(c, d) Galectin-9 secretion in control (NC) or *RAB27A*-depleted (si*RAB27A*#2 and si*RAB27A*#3) HEK-293T cells starved in DMEM for 24 hours was analyzed by immunoblotting (c) and quantified (d). Secretion of indicated proteins in control cells was normalized to 1. Data are presented as mean ± SEM and analyzed using Student's t test (n = 3 independent experiments). ns, not significant.

(e-g) Secretion of indicated proteins in control (NC), *ATG9A*-depleted (si*ATG9A*#1), *RAB8A*-depleted (si*RAB8A*#1), or *RAB27A*-depleted (si*RAB27A*#1) HEK-293T cells after 24 hours of DMEM starvation was analyzed by immunoblotting (e) and quantified (f, g). Secretion of indicated proteins in control cells was normalized to 1. Data are presented as mean ± SEM and analyzed using Student's t test (n = 3 independent experiments). * $p < 0.05$; ** $p < 0.01$; ns, not significant.

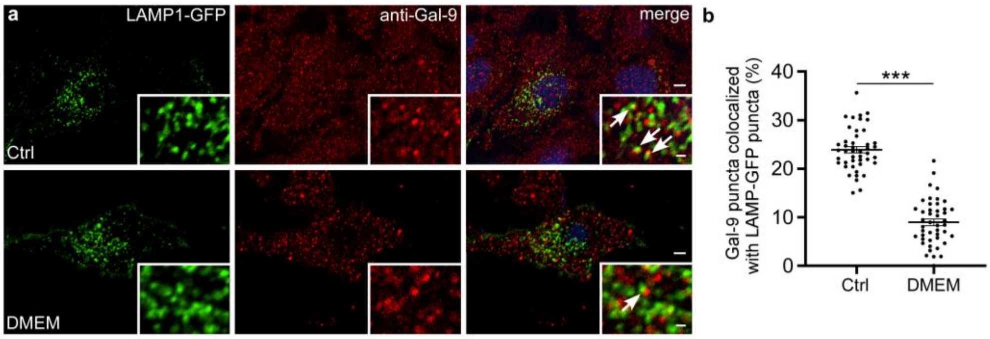

Supplementary Figure 5. Localization of galectin-9 to lysosomes is decreased after DMEM starvation.

(a) Immunostaining of galectin-9 in HeLa cells expressing LAMP1-GFP under normal conditions (Ctrl) or following 24-hour DMEM starvation. The arrows point to galectin-9 puncta colocalized with LAMP1-GFP puncta. Scale bars, 5 μm; inserts, 1 μm.

(b) Quantification of the percentage of galectin-9 puncta colocalized with LAMP1-GFP puncta. Data are presented as mean ± SEM and analyzed using Student's t test (n = 45 cells in each group examined over 3 independent experiments). *** $p < 0.001$.

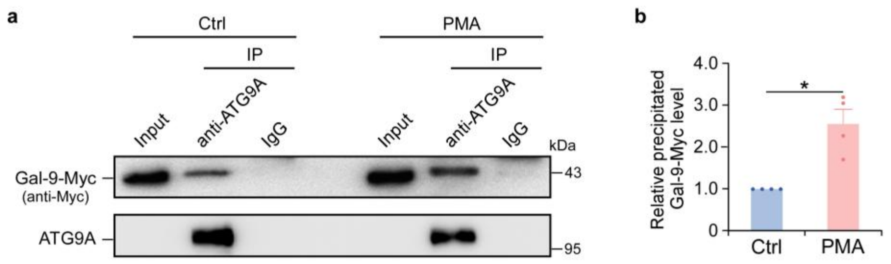

Supplementary Figure 6. Increased interaction between galectin-9 and ATG9A after PMA treatment

(a) HeLa cells expressing galectin-9-Myc were treated with PMA (60 ng/ml). Cell lysates were immunoprecipitated using an anti-ATG9A antibody, followed by immunoblotting with an anti-Myc antibody to detect galectin-9-Myc.

(b) Quantification of galectin-9-Myc levels precipitated by ATG9A (normalized to ATG9A). Data are presented as mean ± SEM and analyzed using Student's t test (n = 4 independent experiments). * $p < 0.05$.

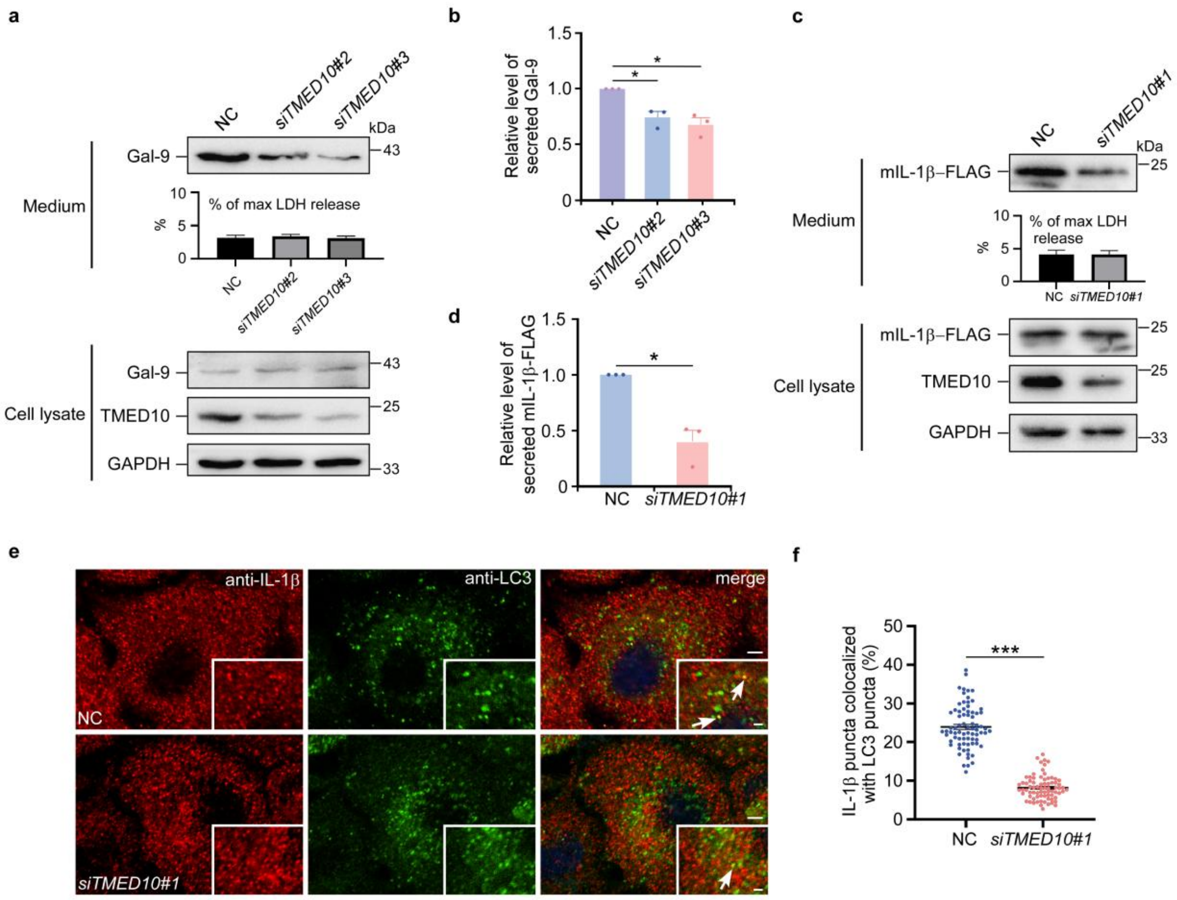

Supplementary Figure 7. TMED10 depletion affects the secretion of both galectin-9 and IL-1β

(a, b) Galectin-9 secretion in control (NC) or *TMED10*-depleted (si*TMED10*#2 and si*TMED10*#3) HEK-293T cells following 24 hours of DMEM starvation was analyzed by immunoblotting (a) and quantified (b). Data are presented as mean ± SEM and analyzed using Student's t test (n = 3 independent experiments). * $p < 0.05$.

(c, d) FLAG-tagged IL-1β secretion in control (NC) or *TMED10*-depleted (si*TMED10*#1) HEK-293T cells following 24 hours of DMEM starvation was analyzed by immunoblotting (c) and quantified (d). Data are presented as mean ± SEM and analyzed using Student's t test (n = 3 independent experiments). * $p < 0.05$.

(e, f) IL-1β and LC3 immunostaining in control (NC) or *TMED10*-depleted (si*TMED10*#1) HeLa cells following 1-hour DMEM starvation. Scale bars, 5 µm; inserts, 1 µm. (e) Representative images were displayed. (f) The percentage of IL-1β

puncta colocalized with LC3-positive puncta was quantified. Data are presented as mean ± SEM and analyzed using Wilcoxon rank sum test (n = 75 cells in each group examined over 3 independent experiments). *** $p < 0.001$.

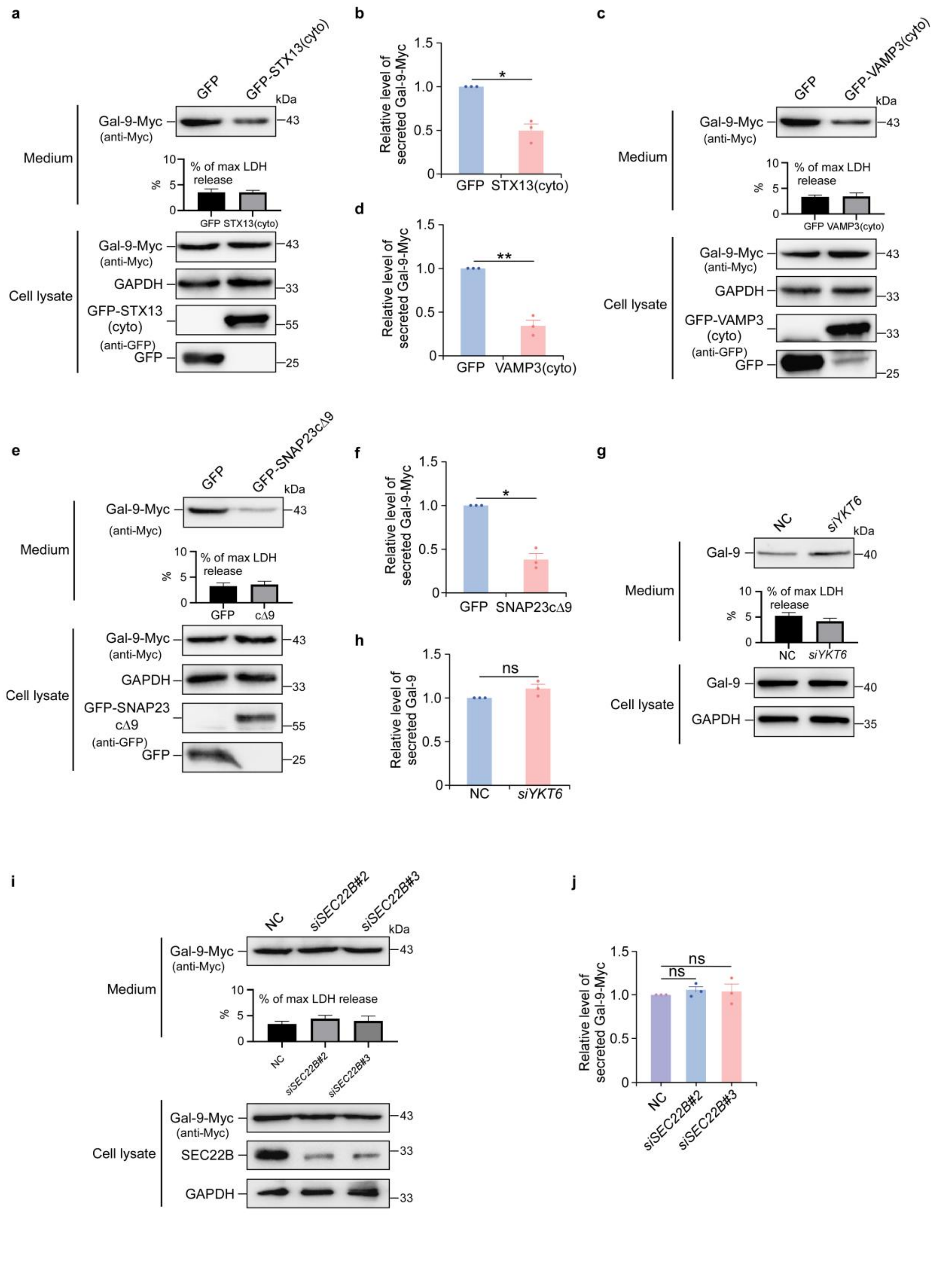

Supplementary Figure 8. The STX13-SNAP23-VAMP3 SNARE complex is involved in galectin-9 secretion

(a, b) Galectin-9-Myc secretion in GFP or GFP-STX13(cyto)-expressing HEK-293T cells following 24 hours of DMEM starvation was analyzed by immunoblotting (a) and quantified (b). Data are presented as mean ± SEM and analyzed using Student's t test (n = 3 independent experiments). * $p < 0.05$.

(c, d) Galectin-9-Myc secretion in GFP or GFP-VAMP3(cyto)-expressing HEK-293T cells following 24 hours of DMEM starvation was analyzed by immunoblotting (c) and quantified (d). Data are presented as mean ± SEM and analyzed using Student's t test (n = 3 independent experiments). ** $p < 0.01$.

(e, f) Galectin-9-Myc secretion in GFP or GFP-SNAP23cΔ9-expressing HEK-293T cells following 24 hours of DMEM starvation was analyzed by immunoblotting (e) and quantified (f). Data are presented as mean ± SEM and analyzed using Student's t test (n = 3 independent experiments). * $p < 0.05$.

(g, h) Galectin-9 secretion in control (NC) or *YKT6*-depleted (si*YKT6*) HeLa cells following 24 hours of DMEM starvation was analyzed by immunoblotting (g) and quantified (h). Data are presented as mean ± SEM and analyzed using Student's t test (n = 3 independent experiments). ns, not significant.

(i, j) Galectin-9-Myc secretion in control (NC) or *SEC22B*-depleted (si*SEC22B*#2 and si*SEC22B*#3) HEK-293T cells following 24 hours of DMEM starvation was analyzed by immunoblotting (i) and quantified (j). Data are presented as mean ± SEM and analyzed using Student's t test (n = 3 independent experiments). ns, not significant.

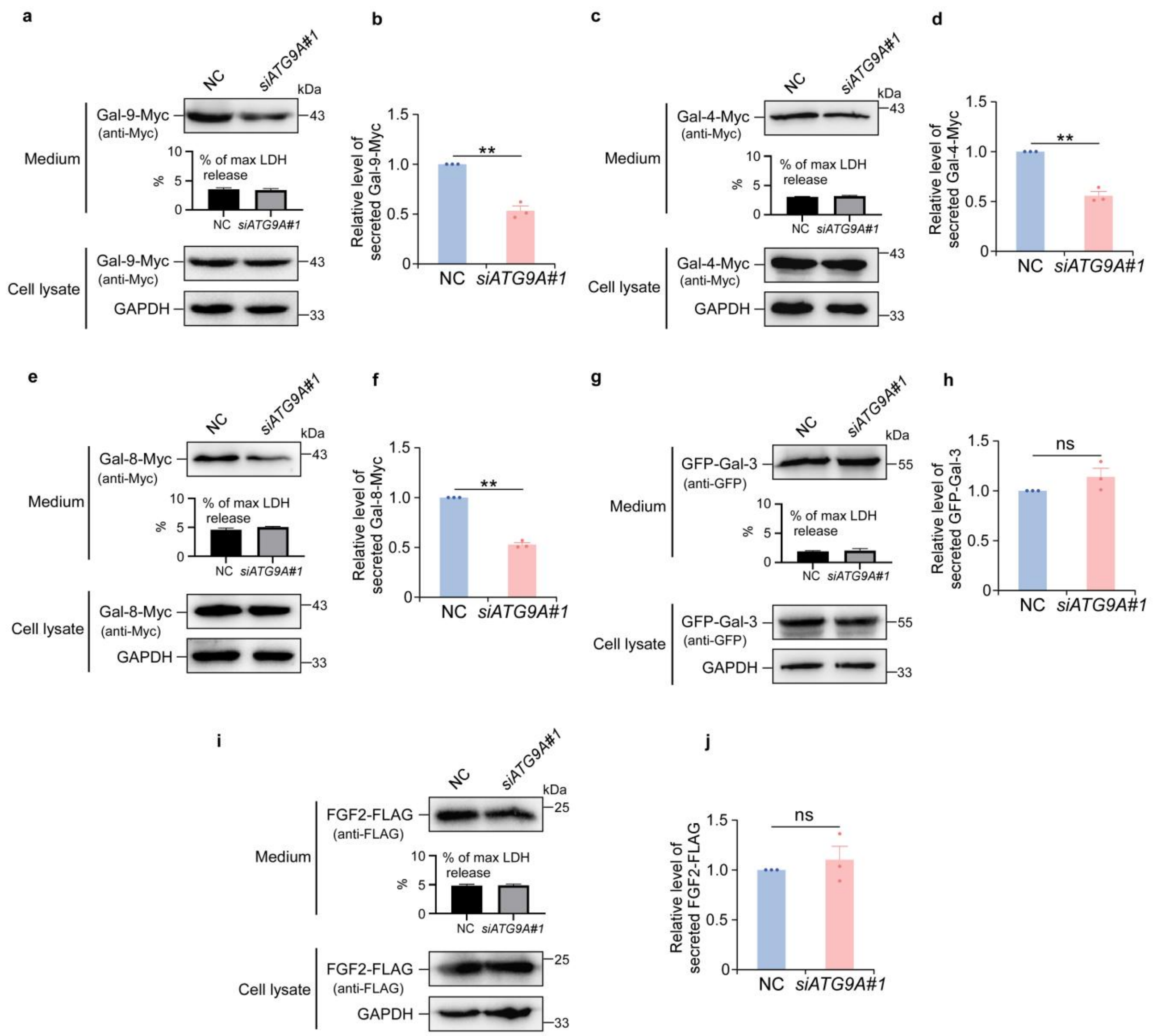

Supplementary Figure 9. The effect of ATG9A on other unconventional secretion cargos in HEK-293T cells

(a, b) Galectin-9-Myc secretion in control (NC) or *ATG9A*-depleted (si*ATG9A*#1) HEK-293T cells following 24 hours of DMEM starvation was analyzed immunoblotting (a) and quantified (b). Data are presented as mean ± SEM and analyzed using Student's t test (n = 3 independent experiments). ** $p < 0.01$.

(c, d) Galectin-4-Myc secretion in control (NC) or *ATG9A*-depleted (si*ATG9A*#1) HEK-293T cells following 24 hours of DMEM starvation was analyzed by

immunoblotting (c) and quantified (d). Data are presented as mean ± SEM and analyzed using Student's t test (n = 3 independent experiments). ** $p < 0.01$.

(e, f) Galectin-8-Myc secretion in control (NC) or *ATG9A*-depleted (si*ATG9A*#1) HEK-293T cells following 24 hours of DMEM starvation was analyzed by immunoblotting (e) and quantified (f). Data are presented as mean ± SEM and analyzed using Student's t test (n = 3 independent experiments). ** $p < 0.01$.

(g, h) GFP-Galectin-3 secretion in control (NC) or *ATG9A*-depleted (si*ATG9A*#1) HEK-293T cells following 24 hours of DMEM starvation was analyzed by immunoblotting (g) and quantified (h). Data are presented as mean ± SEM and analyzed using Student's t test (n = 3 independent experiments). ns, not significant.

(i, j) FGF2-FLAG secretion in control (NC) or *ATG9A*-depleted (si*ATG9A*#1) HEK-293T cells following 24 hours of DMEM starvation was analyzed by immunoblotting (i) and quantified (j). Data are presented as mean ± SEM and analyzed using Student's t test (n = 3 independent experiments). ns, not significant.

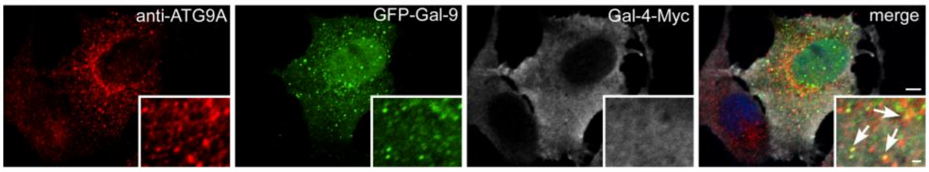

Supplementary Figure 10. Colocalization analysis of ATG9A, galectin-9, and galectin-4

HeLa cells expressing GFP-galectin-9 and galectin-4-Myc were subjected to 24-hour DMEM starvation, followed by immunostaining for ATG9A and galectin-4-Myc and fluorescence imaging. Arrows indicate $ATG9A^{+}GFP\text{-}galectin\text{-}9^{+}$ puncta. Scale bars, 5 μm; inserts, 1 μm. Representative data from three independent experiments are shown.

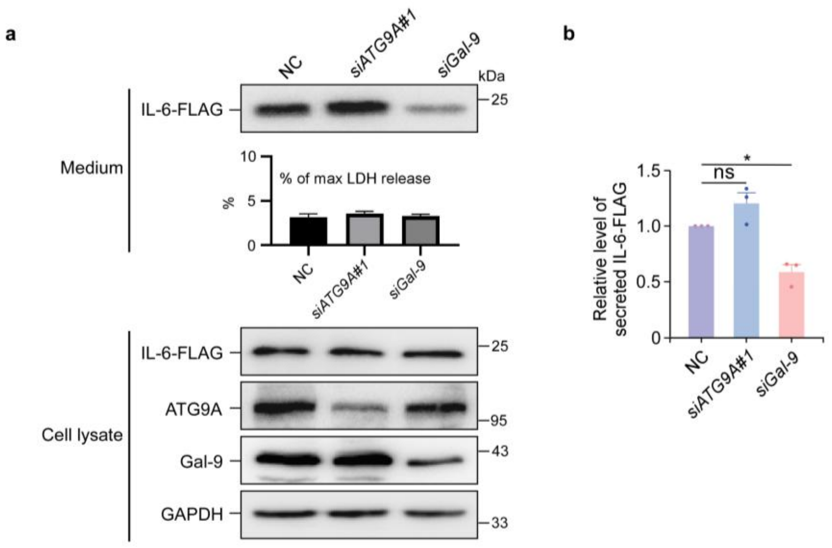

Supplementary Figure 11. Galectin-9, but not ATG9A, is involved in the secretion of IL-6

(a, b) IL-6-FLAG secretion in control (NC), *ATG9A*-depleted (si*ATG9A*#1), or *galectin-9*-depleted (si*Gal-9*) HEK-293T cells following 24 hours of DMEM starvation was analyzed by immunoblotting (a) and quantified (b). Data are presented as mean ± SEM and analyzed using Student's t test (n = 3 independent experiments). * $p < 0.05$; ns, not significant.

Supplementary Table 1. Primer Sequences for Plasmid Construction

Plasmid	Forward Primer	Reverse Primer
pcDNA3.1-*galectin-9*-Myc	TTGGTACCGAGCTCGGATCCATGGCCTTCAGCGGTTCACA	ATGAGCTTTTGCTCCTCGAGTGTCTGCACATGGGTCAGCTG
pcDNA3.1-*galectin-8*-Myc	TTGGTACCGAGCTCGGATCCATGATGTTGTCCTTGAACAACCTA	ATGAGCTTTTGCTCCTCGAGCCAGCTCCTTACTTCCAGCAAG
pcDNA3.1-*galectin-4*-Myc	CCGGAATTCATGGCCTATGTCCCCGC	CCGCTCGAGGATCTGGACATAGGACAAGGTGA
mCherry-*galectin-9*	CCGCTCGAGCTATGGCCTTCAGCGGTTCACA	CTATGTCTGCACATGGGTCAGCTGAATTCCGG
pGEX-5X-2-GST-*galectin-9*	TGATCGAAGGTCGTGGGATCATGGCCTTCAGCGGTTCACA	TCACGATGCGGCCGCTCGAGCTATGTCTGCACATGGGTCAGCT
pcDNA3.1-*ATG9A*-GFP	TAGCGTTTAAACTTAAGCTTATGGCGCAGTTTGACACTGAATAC	CTCACCATGGTGGCCTCGAGTACCTTGTGCACCTGAGGGGGT
pcDNA3.1-*TMED10*-GFP	CGCGGATCCATGTCTGGTTTGTCTGGCCCAC	CCGCTCGAGCTCAATCAATTTCTTGGCCTTGA
pEGFP -*RUSC2*	CGGGATCCCAGGGACAGTGTCTGGTGCTGTTG	CGCTCGAGGTTTTGGCTGCTTCCAGGGGTTG
pEGFP -*STX13*	CGGAATTC T ATGTCATACGGTCCCTTAGA	CGGGATCCTCACTTCGTTTTATAAACTAGC
pEGFP -*VAMP3*	CCCTCGAGCTATGTCTACAGGTCCAACTGC	CGGGATCCTCATGAAGAGACAACCCACA
pEGFP -*SNAP23*	CCCTCGAGCTATGGATAATCTGTCATCAGAAG	CGGGATCCTTAGCTGTCAATGAGTTTCTTT
pEGFP -*SNAP23*-c△9	GCCAATTAAGGATCCACCGGATCTAGATAAC	GGTGGATCCTTAATTGGCAATATCAATACGATCTCTGT
pEGFP -*VAMP3*(cyto)	TTGCAAGTGAGGATCCACCGGATCTAGAT	GTGGATCCTCACTTGCAATTCTTCCACCAATATTTC
pEGFP -*STX13*(cyto)	AAATCTCGCTGAGGATCCACCGGATCTAGAT	GGATCCTCAGCGAGATTTTTCTGATAGTAAGCAG
pcDNA3.1-*ANXA2*-Myc	GATCTGTGTGTGGGGCCCGTTATGGGCCGCCAGCTAG	GATCAGCGGGTGGGCCCTTAGTCATCTCCACCACACAG
pcDNA3.1-*HMGB1*-Myc	AGTGGCGGCCGCTCGAGATGGGCAAAGGAGATCCT	AGCTTTTGCTCCTCGAGTTCATCATCATCATCTTCTTCTT
pEGFP-*galectin-9*	CCCTCGAGCTATGGCCTTCAGCGGTTCCCA	CGGGATCCCTATGTCTGCACATGGGTCAGCT

Supplementary Table 2. siRNA Sequences

siRNA	Sequence
negative control	5'-UUCUCCGAACGUGUCACGUTT-3'
si*ATG9A*#1	5'-CACUCAUGCACUUUGCCAUTT-3'
si*ATG9A*#2	5'-GUCACCUUGGCACCAUAUUTT-3'
si*ATG9A*#3	5'-GUGGACUAUGACAUCCUAUTT-3'
si*STX13*	5'-TCCCTTAGACATGTACCGGAA-3'
si*VAMP3*	5'-TCGGGATTACTGTTCTGGTTA-3'
si*SNAP23*	5'-GGAAGAGAACCUGACUCAATT-3'
si*ULK1*#1	5'-CCUGUGACACAGACGACUUTT-3'
si*ULK1*#2	5'-CAGGAGAAGCCCAUGGAGATT-3'
si*ULK1*#3	5'-CAUCGAGAACGUCACCAAGTT-3'
si*ATG5*	5'-GACGUUGGUAACUGACAAATT-3'
si*LC3*	5'-GAGUGAGAAAGAUGAAGAUTT-3'
si*ATG7*	5'-CCACAGAUGGAGUAGCATT-3'
si*RAB8A*#1	5'-CGCAUUUUUCACUCUCGCCTT-3'
si*RAB8A*#2	5'-CGGAACUGGAUUCGCAACATT-3'
si*RAB8A*#3	5'-CUCGAUGGCAAGAGAAUUATT-3'
si*RAB27A*#1	5'-CGGAUCAGUUAAGUGAAGAAA-3'
si*RAB27A*#2	5'-CCAGUGUACUUUACCAAUATT-3'
si*RAB27A*#3	5'-CAGGAGAGGUUUCGUAGCUTT-3'
si*TMED10*#1	5'-UCACAAGGACCUGCUAGUGTT-3'
si*TMED10*#2	5'-CAGAUUCUGCUGGCCAUAUTT-3'
si*TMED10*#3	5'-CCAACUCGUGAUCCUAGACTT-3'
si*YKT6*	5'-CUAUAAAACUGCCCGGAAA-3'
si*SEC22B*#1	5'-CAGUCGUGCUCGAAGAAAUTT-3'
si*SEC22B*#2	5'-CCUACCAGAUGUACCUUGGTT-3'
si*SEC22B*#3	5'-CCAAGAAGCUCUACAUUGATT-3'
si*STX1*	5'-GGAACACGCGGUAGACUAU-3'
si*STX2*	5'-UAGACAAGCUCUCAAUGAA-3'
si*STX3*	5'-CGACAGGCCUUAAAUGAGA-3'
si*STX4*	5'-GAUCAUUGACUCAACAGAUU-3'
si*STX5*	5'-CGACAACGCAGUGAAUUCATT-3'
si*VTI1A*	5'-GTCGTCCGACTTCGAAGGTTA-3'
si*VTI1B*	5'-CCUUGUGGACCAGCAUCUUTT-3'
si*GOSR1*	5'-CAGAAGAACUGAGCUAUUU-3'
si*GOSR2*	5'-ACGAAUCACUGCAGUUUAA-3'
si*STX8*	5'-AAUGAAACCAGGCGGGUAA-3'
si*STX10*	5'-GGAAGAGACCAUCGGUAUA-3'
si*SNAP29*	5'-GAAGCUAUAAGUACAAGUA-3'
si*VAMP2*	5'-CCCATTAGTTCTTGTATCACA-3'
si*VAMP4*	5'-CAACUUCGAAGGCAAAUGU-3'
si*VAMP7*	5'-CTGCCAAGACAGGATTGTATA-3'
si*SEC22A*	5'-GGGCAAGGCUCCCGAUUAU-3'
si*AP-4*	5'-CGGGAUUCCAGUCCCUCAATT-3'
si*ATG2A*	5'-GCAUUCCCAGUUGUUGGAGUUCCUA-3'
si*ATG2B*	5'-AGGUCUCUCUUGUCUGGCAUCUUUA-3'
