# Supplementary material for: Autophagy-independent role of ATG9A vesicles as carriers for galectin-9 secretion
Source: Nat Commun. 2025 May 7;16:4259. doi: 10.1038/s41467-025-59605-5 (PMC12059159; doi:10.1038/s41467-025-59605-5)
Supplement: Supplementary file 2 — Reporting Summary [file 41467_2025_59605_MOESM2_ESM.pdf]

nature portfolio

Corresponding author(s): Haolin Zhang and Juan Wang

Last updated by author(s): Apr 17, 2025

Reporting Summary

Nature Portfolio wishes to improve the reproducibility of the work that we publish. This form provides structure for consistency and transparency in reporting. For further information on Nature Portfolio policies, see our Editorial Policies and the Editorial Policy Checklist.

Statistics

For all statistical analyses, confirm that the following items are present in the figure legend, table legend, main text, or Methods section.

n/a	Confirmed	
☐	☒	The exact sample size (*n*) for each experimental group/condition, given as a discrete number and unit of measurement
☐	☒	A statement on whether measurements were taken from distinct samples or whether the same sample was measured repeatedly
☐	☒	The statistical test(s) used AND whether they are one- or two-sided<br>*Only common tests should be described solely by name; describe more complex techniques in the Methods section.*
☒	☐	A description of all covariates tested
☐	☒	A description of any assumptions or corrections, such as tests of normality and adjustment for multiple comparisons
☐	☒	A full description of the statistical parameters including central tendency (e.g. means) or other basic estimates (e.g. regression coefficient) AND variation (e.g. standard deviation) or associated estimates of uncertainty (e.g. confidence intervals)
☐	☒	For null hypothesis testing, the test statistic (e.g. *F*, *t*, *r*) with confidence intervals, effect sizes, degrees of freedom and *P* value noted<br>*Give P values as exact values whenever suitable.*
☒	☐	For Bayesian analysis, information on the choice of priors and Markov chain Monte Carlo settings
☒	☐	For hierarchical and complex designs, identification of the appropriate level for tests and full reporting of outcomes
☒	☐	Estimates of effect sizes (e.g. Cohen's *d*, Pearson's *r*), indicating how they were calculated

Our web collection on statistics for biologists contains articles on many of the points above.

Software and code

Policy information about availability of computer code

Data collection	The software for acquiring Western blot images is GelCap ECL (GElCap5.6). The software utilized for capturing confocal images is NIS-Elements Viewer. The software employed for collecting flow cytometry data is FlowJo version X software (Tree Star).
Data analysis	The software used for data analysis is SPSS 25.0 and GraphPad Prism 9.

For manuscripts utilizing custom algorithms or software that are central to the research but not yet described in published literature, software must be made available to editors and reviewers. We strongly encourage code deposition in a community repository (e.g. GitHub). See the Nature Portfolio guidelines for submitting code & software for further information.

Data

Policy information about availability of data

All manuscripts must include a data availability statement. This statement should provide the following information, where applicable:

- Accession codes, unique identifiers, or web links for publicly available datasets
- A description of any restrictions on data availability
- For clinical datasets or third party data, please ensure that the statement adheres to our policy

All data supporting the findings of this study are presented in the paper, Supplementary Information or Source Data file. All data are available upon request. Source data are provided with this paper.

Research involving human participants, their data, or biological material

Policy information about studies with human participants or human data. See also policy information about sex, gender (identity/presentation), and sexual orientation and race, ethnicity and racism.

Reporting on sex and gender	*Use the terms sex (biological attribute) and gender (shaped by social and cultural circumstances) carefully in order to avoid confusing both terms. Indicate if findings apply to only one sex or gender; describe whether sex and gender were considered in study design; whether sex and/or gender was determined based on self-reporting or assigned and methods used.* *Provide in the source data disaggregated sex and gender data, where this information has been collected, and if consent has been obtained for sharing of individual-level data; provide overall numbers in this Reporting Summary. Please state if this information has not been collected.* *Report sex- and gender-based analyses where performed, justify reasons for lack of sex- and gender-based analysis.*
Reporting on race, ethnicity, or other socially relevant groupings	*Please specify the socially constructed or socially relevant categorization variable(s) used in your manuscript and explain why they were used. Please note that such variables should not be used as proxies for other socially constructed/relevant variables (for example, race or ethnicity should not be used as a proxy for socioeconomic status).* *Provide clear definitions of the relevant terms used, how they were provided (by the participants/respondents, the researchers, or third parties), and the method(s) used to classify people into the different categories (e.g. self-report, census or administrative data, social media data, etc.)* *Please provide details about how you controlled for confounding variables in your analyses.*
Population characteristics	*Describe the covariate-relevant population characteristics of the human research participants (e.g. age, genotypic information, past and current diagnosis and treatment categories). If you filled out the behavioural & social sciences study design questions and have nothing to add here, write "See above."*
Recruitment	*Describe how participants were recruited. Outline any potential self-selection bias or other biases that may be present and how these are likely to impact results.*
Ethics oversight	*Identify the organization(s) that approved the study protocol.*

Note that full information on the approval of the study protocol must also be provided in the manuscript.

Field-specific reporting

Please select the one below that is the best fit for your research. If you are not sure, read the appropriate sections before making your selection.

[x] Life sciences [ ] Behavioural & social sciences [ ] Ecological, evolutionary & environmental sciences

For a reference copy of the document with all sections, see nature.com/documents/nr-reporting-summary-flat.pdf

Life sciences study design

All studies must disclose on these points even when the disclosure is negative.

Sample size	All of our quantitative experiments are based on cell lines. The data obtained from cell lines show good consistency and low variation. Therefore, our sample size is determined based on experience.
Data exclusions	No data were excluded from analyses.
Replication	All attempts at replication were successful.
Randomization	Allocation was random.
Blinding	The investigators were not blinded to group allocation during data collection and analysis. The results of the cell experiments are objective data obtained through instrument detection and are not influenced by subjective conditions.

Reporting for specific materials, systems and methods

We require information from authors about some types of materials, experimental systems and methods used in many studies. Here, indicate whether each material, system or method listed is relevant to your study. If you are not sure if a list item applies to your research, read the appropriate section before selecting a response.

Materials & experimental systems

n/a	Involved in the study
☐	☒ Antibodies
☐	☒ Eukaryotic cell lines
☒	☐ Palaeontology and archaeology
☒	☐ Animals and other organisms
☒	☐ Clinical data
☒	☐ Dual use research of concern
☒	☐ Plants

Methods

n/a	Involved in the study
☒	☐ ChIP-seq
☐	☒ Flow cytometry
☒	☐ MRI-based neuroimaging

Antibodies

Antibodies used

Antibodies used in this study: rabbit anti-galectin-9 (abcam, ab69630, 1:1000), rabbit anti-galectin-9 (Thermo Fisher Scientific, PA5-115266, 1:1000), rabbit anti-galectin-9 (abcam, ab227046, 1:1000), goat anti-galectin-9 (AF2045, R&D systems, 1:500), mouse anti-GAPDH (proteintech, 60004-1-Ig, 1:10000), rabbit anti-ATG2A (MBL, PD041, 1:1000), rabbit anti-ATG2B (proteintech, 25155-1-AP, 1:1000), rabbit anti-ATG9A (MBL, PD042, 1:1000 for western blot and 1:200 for immunofluorescence staining), mouse anti-GST (proteintech, 66001-2-Ig, 1:2000), rabbit anti-Myc (proteintech, 16286-1-AP, 1:2000 for western blot and 1:200 for immunofluorescence staining), rabbit anti-GFP (proteintech, 50430-2-AP, 1:2000), mouse anti-annexin A6 (Santa Cruz Biotechnology, sc-271859, 1:1000), mouse anti-actin (proteintech, 66009-1-Ig, 1:2000), mouse anti-V5 (Thermo Fisher Scientific, R960-25, 1:500), rabbit anti-FLAG (proteintech, 20543-1-AP, 1:10000), rabbit anti-IL-1β (abcam, ab9722, 1:1000 for western blot and 1:200 for immunofluorescence staining), rabbit anti-LC3B (Cell Signaling Technology, 2775S, 1:1000), Mouse monoclonal anti-LC3 (MBL, M152-3, 1:200), mouse anti-TMED10 (Proteintech, 67876-1-Ig, 1:1000), rabbit anti-RAB27A (Proteintech, 17817-1-AP, 1:1000), rabbit anti-RAB8A （Proteintech, 55296-1-AP, 1:1000), rabbit anti-SEC22B (Proteintech, 14776-1-AP, 1:1000), rabbit anti-ULK1(Sigma, A7481, 1:1000), mouse anti-HNRNPK (Proteintech, 67708-1-Ig, 1:1000), FITC AffiniPure Goat anti-Mouse IgG (H+L) (Jackson, 115-095-003, 1:400), Alexa Fluor® 647 AffiniPure™ Donkey Anti-Mouse IgG (H+L) (Jackson, 715-605-150, 1:400), Rhodamine AffiniPure Goat anti-Rabbit IgG (Jackson, 111-025-003, 1:400), Rhodamine AffiniPure Goat anti-Mouse IgG (Jackson, 115-025-003, 1:400), HRP-conjugated Affinipure Goat Anti-Rabbit IgG (H+L) (proteintech, SA00001-2, 1:5000), HRP-conjugated Affinipure Goat Anti-mouse IgG (H+L) (proteintech, SA00001-1, 1:5000), mouse anti-Biotin (Santa Cruz Biotechnology, sc-53179, 1:500).

Validation

Rabbit anti-galectin-9 (abcam, ab69630) has been cited in 31 publications, underscoring its widespread application and reliability in relevant research fields. Rabbit anti-galectin-9 (Thermo Fisher Scientific, PA5-115266) is a polyclonal antibody designed for the detection of galectin-9 in various applications. This antibody is produced in rabbits and demonstrates high specificity and sensitivity. It has been validated for use in techniques such as Western blot, immunohistochemistry, and immunofluorescence. PA5-115266 is supplied in a stabilized buffer to ensure long-term performance and reliability. Rabbit anti-galectin-9 (abcam, ab227046) has been cited in 6 publications, underscoring its widespread application and reliability in relevant research fields. Goat anti-galectin-9 (AF2045, R&D systems) has been cited in 22 publications, underscoring its widespread application and reliability in relevant research fields. Mouse anti-GAPDH (proteintech, 60004-1-Ig) has been cited in 12226 publications, underscoring its widespread application and reliability in relevant research fields. Rabbit anti-ATG2A (MBL, PD041), rabbit anti-ATG2B (proteintech, 25155-1-AP), and rabbit anti-ATG9A (MBL, PD042) have been widely applied and proven reliable in relevant research fields. Mouse anti-GST (proteintech, 66001-2-Ig) has been cited in 138 publications, underscoring its widespread application and reliability in relevant research fields. Rabbit anti-Myc (proteintech, 16286-1-AP) has been cited in 410 publications, underscoring its widespread application and reliability in relevant research fields. Rabbit anti-GFP (proteintech, 50430-2-AP) antibody has been cited in 738 publications, underscoring its widespread application and reliability in relevant research fields. Mouse anti-annexin A6 (Santa Cruz Biotechnology, sc-271859) has been cited in 8 publications, underscoring its widespread application and reliability in relevant research fields. Mouse anti-actin (proteintech, 66009-1-Ig) has been cited in 6934 publications, underscoring its widespread application and reliability in relevant research fields. Mouse anti-V5 (Thermo Fisher Scientific, R960-25) has been cited in 1217 publications, underscoring its widespread application and reliability in relevant research fields. Rabbit anti-FLAG (proteintech, 20543-1-AP) has been cited in 1080 publications, underscoring its widespread application and reliability in relevant research fields. Rabbit anti-IL-1β (abcam, ab9722) has been cited in 864 publications, underscoring its widespread application and reliability in relevant research fields. Rabbit anti-LC3B (Cell Signaling Technology, 2775S) polyclonal antibody is produced by immunizing animals with a synthetic peptide corresponding to residues near the amino terminus of LC3B. Antibodies were purified by peptide affinity chromatography. Mouse monoclonal anti-LC3 (MBL, M152-3) has been cited in 2 publications, underscoring its widespread application and reliability in relevant research fields. Mouse anti-TMED10 (Proteintech, 67876-1-Ig) is a monoclonal antibody designed for the detection of TMED10 in various applications. This antibody is produced in mice and demonstrates high specificity and sensitivity. It has been validated for use in techniques such as Western blot, immunohistochemistry, and immunofluorescence. 67876-1-Ig is supplied in a stabilized buffer to ensure long-term performance and reliability. Rabbit anti-RAB27A (Proteintech, 17817-1-AP) has been cited in 61 publications, underscoring its widespread application and reliability in relevant research fields. Rabbit anti-RAB8A （Proteintech, 55296-1-AP) has been cited in 27 publications, underscoring its widespread application and reliability in relevant research fields. Rabbit anti-SEC22B (Proteintech, 14776-1-AP) has been cited in 5 publications, underscoring its widespread application and reliability in relevant research fields. Rabbit anti-FLAG (Proteintech, 20543-1-AP) has been cited in 1080 publications, underscoring its widespread application and reliability in relevant research fields. Rabbit anti-ULK1(Sigma, A7481) has been cited in 100 publications, underscoring its widespread application and reliability in relevant research fields. Mouse anti-HNRNPK (Proteintech, 67708-1-Ig) has been validated for use in several common laboratory techniques, including Western blot, immunohistochemistry, and immunofluorescence. The antibody is supplied in a stabilized buffer to maintain its performance and reliability over time. This makes it a reliable tool for researchers studying HNRNPK in various experimental contexts.

Eukaryotic cell lines

Policy information about cell lines and Sex and Gender in Research

Cell line source(s)	HeLa (CVCL_0030), HEK-293T (CVCL_0063), MEF (CVCL_C1M8), and THP-1 (CVCL_0001) cells were obtained from ATCC (Manassas, VA, USA). ATG9A knockout and FIP200 knockout HeLa cells, as well as Atg5 knockout MEF cells, were generously provided by Dr. Hong Zhang from the Institute of Biophysics, Chinese Academy of Sciences.
Authentication	Cell line authentication was performed in the past three years. Authentication was done using short tandem repeat (STR) profiling, which involved amplifying specific DNA regions and comparison against known cell line profiles.
Mycoplasma contamination	The cultures were regularly monitored for mycoplasma contamination using PCR-based assays to confirm that the cell lines remained free from mycoplasma. All experiments were performed with mycoplasma-free cells.
Commonly misidentified lines (See ICLAC register)	HEp-2, Intestine 407, and KB cells are among the most misidentified cell lines associated with HeLa cells. HEK293FT, HEK293S, and BOSC23 cells are among the most misidentified cell lines associated with HEK-293T cells. OP9, C2C12, NIH 3T3, and STO Cells are among the most misidentified cell lines associated with MEF cells. U-937, Mono Mac 6, and MUTZ-3 cells are among the most misidentified cell lines associated with THP-1 cells. We acknowledge that the cell lines we used are commonly misidentified and confirm their identity through STR profiling.

Plants

Seed stocks	*Report on the source of all seed stocks or other plant material used. If applicable, state the seed stock centre and catalogue number. If plant specimens were collected from the field, describe the collection location, date and sampling procedures.*
Novel plant genotypes	*Describe the methods by which all novel plant genotypes were produced. This includes those generated by transgenic approaches, gene editing, chemical/radiation-based mutagenesis and hybridization. For transgenic lines, describe the transformation method, the number of independent lines analyzed and the generation upon which experiments were performed. For gene-edited lines, describe the editor used, the endogenous sequence targeted for editing, the targeting guide RNA sequence (if applicable) and how the editor was applied.*
Authentication	*Describe any authentication procedures for each seed stock used or novel genotype generated. Describe any experiments used to assess the effect of a mutation and, where applicable, how potential secondary effects (e.g. second site T-DNA insertions, mosiacism, off-target gene editing) were examined.*

Flow Cytometry

Plots

Confirm that:

- [x] The axis labels state the marker and fluorochrome used (e.g. CD4-FITC).
- [x] The axis scales are clearly visible. Include numbers along axes only for bottom left plot of group (a 'group' is an analysis of identical markers).
- [x] All plots are contour plots with outliers or pseudocolor plots.
- [x] A numerical value for number of cells or percentage (with statistics) is provided.

Methodology

Sample preparation	Single-cell suspensions were stained with specific primary antibodies or isotype controls as negative controls for 30 min at 4 ° C. The following primary antibody was used: goat anti-galectin-9 (AF2045, R&D Systems) at a concentration of 20 µg/ml. Secondary antibody conjugated to Alexa Fluor 555 (Abcam, ab150134) was used at a 1:100 (v/v) dilution. All antibody incubations were carried out in PBS supplemented with 2% fetal bovine serum.
Instrument	Flow cytometric analysis was performed using a FACSVerse™ instrument (BD Biosciences).
Software	Data were analyzed using FlowJo version X software (Tree Star).
Cell population abundance	100, 000 cells were used for each biological repeat.
Gating strategy	The qualified cells for fluorescent analyses were filtered by forward-scattered light (FSC) and side-scattered light (SSC). The gating strategy was designed to define the boundaries between "positive" and "negative" staining cell populations based on fluorescence intensity, using appropriate isotype controls and/or unstained samples to set the threshold.

- [ ] Tick this box to confirm that a figure exemplifying the gating strategy is provided in the Supplementary Information.
